# Relevance of glycosylation of S-layer proteins for cell surface properties

**DOI:** 10.1016/j.actbio.2015.03.020

**Published:** 2015-06

**Authors:** Bernhard Schuster, Uwe B. Sleytr

**Affiliations:** a Institute for Synthetic Bioarchitectures, Department of NanoBiotechnology, University of Natural Resources and Life Sciences, Vienna, Muthgasse 11, 1190 Vienna, Austria; b Institute for Biophysics, Department of NanoBiotechnology, University of Natural Resources and Life Sciences, Vienna, Muthgasse 11, 1190 Vienna, Austria

**Keywords:** Biomimetics, S-layer (glyco)proteins, Glycans, Hydration layer, General features of S-layers

## Abstract

Elucidating the building principles and intrinsic features modulating certain water-associated processes (*e.g.*, surface roughness in the nanometer scale, surface hydration and accompanied antifouling property, *etc*.) of surface structures from (micro)organisms is nowadays a highly challenging task in fields like microbiology, biomimetic engineering and (bio)material sciences. Here, we show for the first time the recrystallization of the wild-type S-layer glycoprotein wtSgsE from *Geobacillus stearothermophilus* NRS 2004/3a and its recombinantly produced non-glycosylated form, rSgsE, on gold sensor surfaces. Whereas the proteinaceous lattice of the S-layer proteins is forming a rigid layer on the sensor surface, the glycan chains are developing an overall soft, highly dissipative film. Interestingly, to the wtSgsE lattice almost twice the amount of water is bound and/or coupled in comparison with the non-glycosylated rSgsE with the preferred region being the extending glycan residues. The present results are discussed in terms of the effect of the glycan residues on the recrystallization, the adjoining hydration layer, and the nanoscale roughness and fluidic behavior. The latter features may turn out to be one of the most general ones among bacterial and archaeal S-layer lattices.

## Graphical abstract

**Figure:**
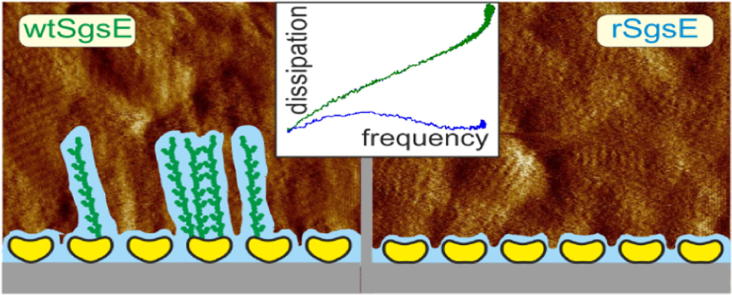

## 1 Introduction

One of the most fascinating cell envelope structures in prokaryotic organisms is two dimensional arrays of protein or glycoprotein subunits, termed S-layers [1]. The widespread occurrence as one of the most common envelope surface structures in archaea and bacteria and the high physiological expense of S-layers raise the question, which selection advantage has S-layer carrying organisms in their natural and frequently highly competitive habitats. Moreover, the exploitation of S-layers as part of more complex supramolecular structures, particularly as patterning element for nanobiotechnological applications and in synthetic biology is a rapidly developing field [1–3].

Bacterial S-layers are highly porous protein mesh works with a unit cell size in the range of 3–30 nm, a thickness of 5–10 nm, and an estimated porosity of approximately 70%. In many S-layer lattices two or more distinct classes of pores in the range of approximately 2–8 nm have been identified. The planar assemblies of identical protein or glycoprotein subunits can be aligned in lattices with oblique (p1, p2), square (p4), or hexagonal (p3, p6) symmetry [1–7]. So far no general biological function has been found and many of the functions assigned to S-layers, for instance, isoporous and/or protective coating of the cell, surface recognition and cell adhesion to substrates, receptor–substrate interactions, templated fine-grain mineralization, as well as mediation of pathogenicity-related phenomena still remain hypothetical [1,8,9].

In addition to unique physicochemical surface properties, the repetitive topographical characteristics of S-layers should be considered as relevant feature affecting hydrodynamic surface properties of cells. It is tempting to speculate that the defined roughness of S-layer surfaces determines the flow resistance of cells in natural environments in a similar way. Studies on friction flows of liquids at nanopatterned interfaces have shown that the slippage of fluids at channel boundaries is greatly increased by using surfaces that are patterned on the nanometer scale [10].

Another important feature of S-layer proteins is that many of them comprise of glycans, the carbohydrate moieties of glycoproteins exposed on the cell surface [11–13]. The cell envelope may exclusively comprise of S-layer glycoproteins or, as in the case of *Geobacillus stearothermophilus* NRS 2004/3a which was investigated in the present study, of an intermixture of S-layer proteins and glycoproteins with different glycosylation patterns [14,15]. From a general point of view, S-layer glycoproteins have been implicated in a multitude of cellular processes, including immune response, intracellular targeting, intercellular recognition, and protein folding and stability [5,16]. Not only the presence of carbohydrate moieties but also the density of glycan display, glycan heterogeneity and molecular scale precision of interaction are most relevant aspects [12,17,18]. Only little is known about structure–function relationships of S-layer glycoproteins. However, one may speculate that surface-exposed glycans may improve the flagella-driven mobility of glycoprotein-carrying microorganisms in complex, natural habitats (*e.g.*, soil, mud, sediments, glycocalyx, biofilms, body fluids) [19].

In general, two major classes of antifouling materials, namely polyhydrophilic and polyzwitterionic materials have been figured out [20]. It is hypothesized that the antifouling ability of materials is tightly correlated with a hydration layer near the surface [21], because a tightly bound water layer forms a physical and energetic barrier to prevent protein adsorption on the surface. Water molecules residing on and/or penetrating into antifouling materials can be classified into two types of “surface-bound” waters formed by (1) hydrogen bonding for hydrophilic materials and by (2) even more strongly ionic solvation for zwitterionic materials [20,22]. Furthermore, spatially controlled binding, *i.e.*, patterning, of bioactive molecules on designated regions of solid surfaces is of great importance for the design of antifouling, bioactive surfaces for, *e.g.*, diagnostics and sensors [23]. In this context it is interesting to note that an excellent antifouling property of S-layers can also be deduced from the perfectly clean surfaces of bacterial cells seen in TEM micrographs of freeze-etched preparations [1,24–26]. Even when cells were harvested from complex environments or growth media containing macromolecular components the S-layer lattices were never masked by adsorbed molecules like, *e.g.*, proteins. Most recently the unique antifouling and cytophobic properties of S-layers were successfully exploited for the coating of microfluidic channels in lab-on-a-chip devices [27,28].

Moreover, native S-layer proteins from several *Bacillus* strains are comprised of carboxyl groups, which are neutralized by an equal number of amino groups and thus, leading to a charge neutral outer surface [29–31]. In addition to these polyzwitterionic characteristics it is self-evident that crystalline S-layer lattices are highly polyhydrophilic because water molecules may penetrate and lock to form a hydrogen–bond network in the pores and bonds to groups on the protein surface [32]. Moreover, hydration of S-layer glycoproteins may even be increased by water-exposed, highly hydrophilic glycan residues. Thus, S-layer lattices in general may be considered as highly hydrated, ultrathin biological antifouling materials.

Considering the combination of antifouling properties (including a hydration layer adjoining the surface), increased slippage and higher capillary permeability, the presence of S-layers may facilitate flagella-driven cell locomotion in natural habitats [19]. Interestingly, a repeated change between relocation and adhesion was described for archaea [33]. This relocate-and-seek behavior would enable the cells to seek for and remain in a favorable surrounding and would, therefore, be of great advantage for them. These features may justify the energy expense of S-layer protein synthesis and may turn out to be one of the most general ones among bacterial and archaeal S-layers [1].

**Fig. 1:**
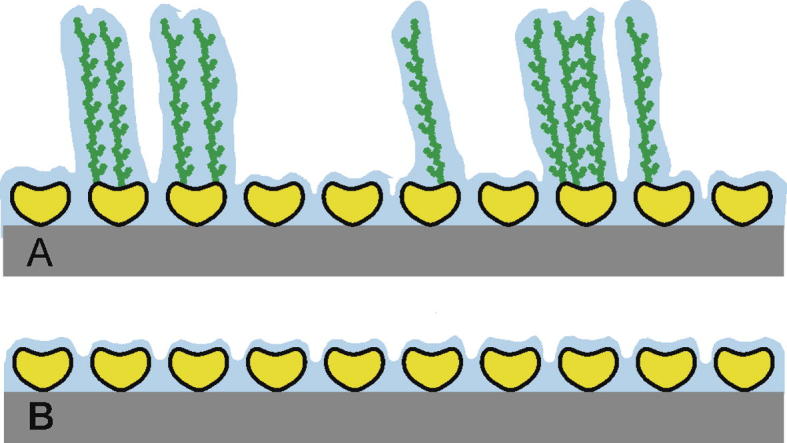
Schematic drawing of the recrystallized S-layer (glyco)proteins SgsE from *Geobacillus stearothermophilus* NRS 2004/3a. In (A) the recrystallized S-layer glycoprotein wtSgsE (yellow) with branched, fully extended glycan moieties (green) and in (B) the recrystallized S-layer protein rSgsE (yellow) are shown. The intermediary liquid locked-in by the pores within the S-layer lattice and the adjoining, bound water shell is indicated in blue. The scheme is not drawn to scale.

In the present study, we have exploited for the first time, the relevance of glycosylation at its “native” condition, where a mixture of (glycosylated) S-layer proteins completely covered the surface of the ubiquitous organism *G. stearothermophilus* NRS 2004/3a, which is not specialized for specific habitats. The recrystallization characteristics and surface properties (i.e., surface hydration, nanoscale fluidic behavior) of this so-called wild-type SgsE (wtSgsE) glycoprotein (schematically depicted in Fig. 1A) are compared to the recombinantly produced protein SgsE (rSgsE), which is N-terminally truncated by 130 amino acids and is lacking the covalently linked carbohydrate moiety (Fig. 1B) [34]. This truncated form has been chosen because (1) the broadest knowledge has accumulated for this non-glycosylated counterpart of wtSgsE, (2) identical S-layer lattice formation compared to wild-type (oblique symmetry, *a* = 11.6 nm, *b* = 9.4 nm, and *γ* ≈ 78° [35]), and (3) highest yield of recombinant protein production [34]. Herein we show that wtSgsE and rSgsE reveal very similar and laterally homogeneous morphology as determined by atomic force microscopy (AFM). Surface plasmon resonance (SPR) spectroscopy and quartz crystal microbalance with dissipation monitoring (QCM-D) are used to elucidate qualitative differences in the adsorption and self-assembly process, the final mass deposited per unit area, and the coupled and bound water within and on the lattice formed by the glycoprotein wtSgsE and the protein rSgsE, respectively.

## 2 Experimental

Unless otherwise stated, all solvents and reagents were purchased from Sigma–Aldrich (Vienna). Buffer solutions were prepared with Milli-Q water (resistivity: ⩾18.2 MΩ cm^−1^).

### 2.1 Bacterial strain and growth conditions

*G. stearothermophilus* NRS 2004/3a was obtained from the N. R. Smith Collection, US Department of Agriculture (Peoria, IL) and was grown in a fermenter on modified S-VIII medium at 55 °C as previously described [35]. Cells were separated from culture broth by continuous centrifugation (Sepatech 17 RS centrifuge; Heraeus, Vienna, Austria) at 16,000×*g* and 4 °C. The biomass was stored at −20 °C.

### 2.2 Preparation of the wild-type S-layer glycoprotein samples

Wild-type S-layer glycoprotein was isolated from cell wall preparations by extraction with 5 M guanidine hydrochloride (GHCl; Fluka, Buchs, Switzerland) according to a previously described procedure [35]. Purification of the wild-type protein wtSgsE was performed and monitored by sodium dodecyl sulfate polyacrylamide gel electrophoresis (SDS–PAGE; see Supplementary data, Fig. S1) as previously described [36].

### 2.3 Preparation of the recombinant protein

All experiments were carried out with a 130 amino acid N-terminal truncation (rSgsE_131–903_) [34] of the S-layer protein SgsE of *G. stearothermophilus* NRS 2004/3a. In the following, rSgsE_131–903_ is called rSgsE for simplification.

*Escherichia coli* DH5a (Invitrogen, Lofer, Austria) was used for cloning; overexpression of proteins was accomplished in *E. coli* BL21 Star (DE3). Both *E. coli* strains were grown at 37 °C in Luria–Bertani broth (LB broth) supplemented with kanamycin (50 mg mL^−1^). Overexpression and purification of the recombinant protein were performed and monitored by SDS–PAGE (data not shown) as previously described [34]. Protein concentration was determined by the Bio-Rad protein assay (Bio-Rad, Vienna, Austria) using BSA as standard. The molecular weight of rSgsE was calculated to be 82,800 Da [37].

### 2.4 Atomic force microscopy (AFM)

A Digital Instruments Nanoscope IIIa (Santa Barbara, CA, USA) was used with a J-scanner (maximal scan size, 130 mm). Standard 200 mm long oxide-sharpened silicon nitride cantilevers (NanoProbes, Digital Instruments) with a nominal spring constant of 0.06 N m^−1^ were used for imaging. The S-layer proteins on the QCM-D quartz crystals were imaged by AFM at a scan rate between 4 and 5 Hz. The applied force was kept to a minimum during scanning to prevent the tip from modifying the sample surface. Scanning was carried out in contact mode (deflection and height images) in a liquid cell under saline solution (100 mM NaCl) to reduce electric repulsion between tip and sample. The samples were stored at 6 °C for up to 12 h before the microscopical investigation was started. AFM images were treated using the WSxM program [38].

### 2.5 Surface plasmon resonance (SPR) measurements

SPR-gold wafers (Ssens, Hengelo, Netherlands) were cleaned by UV/ozone treatment (Plasma Prep2, Gala, Gabler Labor Instruments, Germany). SPR measurements were performed using a Biacore 2000 system (BIACORE AB, Uppsala, Sweden). If not otherwise stated, all experiments were carried out with an S-layer (glyco)protein concentration of 50 μg mL^−1^ and with a continuous flow of 5 mL min^−1^ at 25 °C.

### 2.6 Quartz crystal microbalance with dissipation monitoring (QCMD) measurements

QCM-D measurements were carried out with a QE401 (electronic unit)/QFM401 (flow module) instrument from Q-sense AB (Gothenburg, Sweden). The QCX301 gold crystals (Q-Sense AB, Gothenburg, Sweden) were cleaned before the measurement by immersion in a 5:1:1 (vol/vol) solution of H_2_O:NH_3_(25%):H_2_O_2_(30%) at 75 °C for 10 min followed by rinsing with Milli-Q water and drying in a stream of nitrogen gas. Before any measurements, the crystals were UV/ozone treated. Frequency (Δf) and dissipation (ΔD) shifts were recorded using Q-Tools v3.1.25 software from Q-Sense. The presented results correspond to the 5^th^ overtone. All experiments were done at a temperature of 25 °C ± 0.02 °C. Except measurements studying the concentration dependent adsorption rates for wtSgsE and rSgsE, the S-layer protein concentration was 50 μg mL^−1^. Further steps for detailed evaluation of the QCM-D data are described in the Supplementary data section.

## 3 Results and discussion

### 3.1 Estimation of the composition of the glycoprotein wtSgsE

The analysis of the composition of the wtSgsE resulted in 66.2 ± 5.1% non-glycosylated, 24.2 ± 3.6% carrying one glycan chain, 7.7 ± 1.9% carrying two glycan chains, and 1.9 ± 1.3% carrying three glycan chains (see Supplementary data, Fig. S1). With these data and the average mass of the homopolymeric l-rhamnan found in wtSgsE [36,39], it could be estimated that the glycan content accounts for approximately 3.3% of the molecular mass of wtSgsE and consequently 96.7% accounts for S-layer protein. The mean molecular weight of the used wtSgsE was calculated to be 97,670 Da, whereas 82,800 Da has been reported for rSgsE (Table 1) [37]. The degree of glycosylation of the S-layer protein wtSgsE (3.3%) is in good accordance with previously published data where it has been reported that the degree of glycosylation of bacterial S-layer proteins, that is, the covalent *O*-glycosidic linkage of glycan moieties to select serine, threonine, and tyrosine residues, varies generally between 2% and 10% (w/w) [12].

The molecular length of the branched, fully extended homopolymeric glycan chain composed of 15 tri-rhamnose repeating units was estimated to be roughly 32 nm, a length approximately 4 times higher than the thickness of the wtSgsE monolayer (see Supplementary data). This value is also in good accordance with ferritin labeling of the glycan chains of the S-layer glycoprotein from *Clostridium thermohydrosulfuricum* L111–69 [12,13].

### 3.2 AFM measurements

**Fig. 2:**
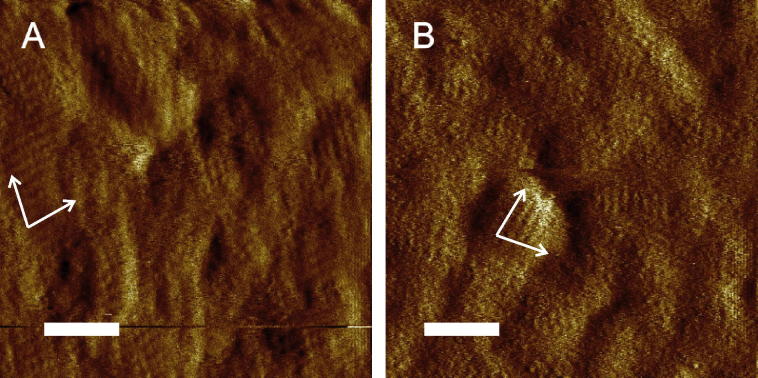
AFM images of the recrystallized S-layer protein from *Geobacillus stearothermophilus* NRS 2004/3a. In (A) the S-layer glycoprotein wtSgsE and in (B) the S-layer protein rSgsE are shown. Base vectors reveal identical orientation of the oblique lattice with the exposed outer face regarding the orientation of the S-layer lattice on the cell. The bars correspond to 100 nm. The (glyco)protein concentration was in both cases 50 μg mL^−1^.

High-resolution AFM images and measurements by surface sensitive methods like SPR and QCM-D are valuable tools not only to investigate S-layer recrystallization but also protein self-assembly at the nanoscale in general. In the present study, the morphology of the recrystallized monomolecular wtSgsE and rSgsE lattices was investigated directly on gold-coated SPR and QCM-D sensor surfaces by AFM. This is important because the coverage of the sensor area by the S-layer proteins is necessary to evaluate both, the SPR and QCM-D data. As depicted in Fig. 2, both S-layer proteins showed the well-known oblique p2 lattice symmetry with an identical orientation [35,40]. For both proteins, not only the size of the single crystallites forming a coherent layer on the sensor surface, but also the smoothness of the S-layer lattices did not differ significantly. Unfortunately, the gold-coated sensor surfaces are not as flat as mica or silicon wafers and hence, it was not possible to determine the lattice parameters from the AFM images. To conclude, S-layer lattices of wtSgsE and rSgsE with a very similar morphology, which can be treated in the SPR and QCM-D experiments as laterally homogeneous [41], have been self-assembled on the sensor surfaces (Fig. 2). These results are in accordance with a previously published study where evidence has been provided that deleting 130 amino acids, or even 330 amino acids from the N-terminus of SgsE apparently does not influence the S-layer lattice formation. In other words, the self-assembly and lattice parameters of truncated, recombinantly produced rSgsEs were identical to those reported for wtSgsE with the advantage of giving the highest yield of recombinant protein production [34].

### 3.3 SPR measurements

**Fig. 3:**
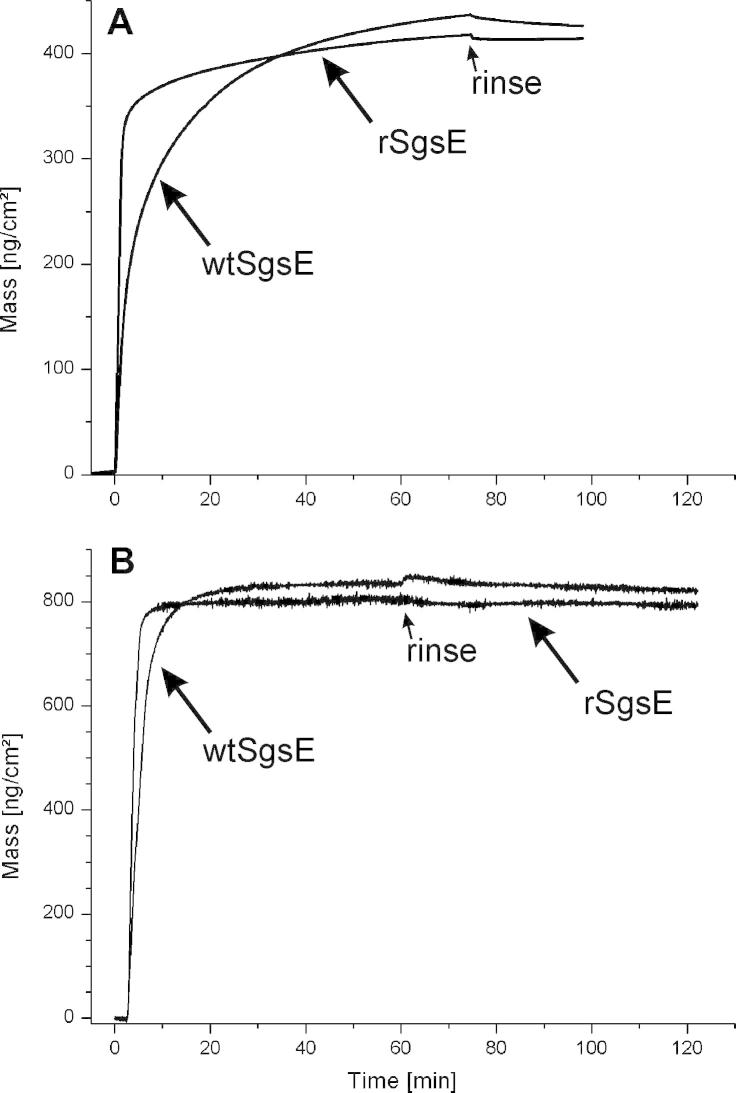
Recrystallization process of two different S-layer (glyco)proteins from *Geobacillus stearothermophilus* NRS 2004/3a (protein concentration of 50 μg mL^−1^) on gold-covered sensor surfaces. The increase in mass as a function of time measured by (A) surface plasmon resonance (SPR) spectroscopy and (B) quartz crystal microbalance with dissipation monitoring (QCM-D) is shown for the S-layer glycoprotein wtSgsE and S-layer protein rSgsE. The biomolecular surface was controlled to form a coherent layer at final mass areal density by AFM.

The adsorption and subsequent recrystallization of the S-layer (glyco)proteins wtSgsE and rSgsE was followed by SPR over a period of approximately 100 min. A clear increase in mass was determined for both S-layers which finally reached a maximum. Subsequent rinsing caused no significant detachment of the formed S-layer lattices. Looking at the sensorgrams, the difference between the glycoprotein wtSgsE and the protein rSgsE was clearly detectable both, in the increase in areal mass density per time (kinetics) and the final maximal mass attached on the sensor surface (Fig. 3A). The increase in mass for rSgsE was much faster and after approximately the first three minutes more than 85% of the final areal mass density of 402.5 ng cm^−2^ was attached to the sensor surface (Table 2). In contrast, the increase in mass was slower for the glycoprotein wtSgsE and there was an exponential approximation to the final areal mass density of 439.2 ng cm^−2^ (Table 2). Finally, from these data it can be concluded that after approximately 60–70 min the self-assembly and recrystallization process forming the monomolecular array have been completed.

The difference of the areal mass density determined by SPR between wtSgsE and rSgsE was calculated to be 36.7 ng cm^−2^ (Table 2). This mass can be attributed to the mass of the glycan chains plus the difference in protein mass of wtSgsE and rSgsE as the latter is truncated by 130 amino acids compared to the protein part of wtSgsE. Taking the molecular weight of wtSgsE and rSgsE into consideration and assuming the same lattice constants for both S-layer proteins (lattice parameters: *a* = 11.6 nm, *b* = 9.4 nm, *γ* ≈ 78°) [18,34,35], this difference in areal mass density is roughly in accordance with the theoretically calculated one of 46.5 ng cm^−2^.

### 3.4 QCM-D measurements

The adsorption and subsequent recrystallization of wtSgsE and rSgsE was also followed by QCM-D over a period of approximately 120 min (Fig. 3B). A clear increase in mass was determined for both S-layers but with this method the final maxima were reached after approximately 10 min and 25 min for rSgsE and wtSgsE, respectively. Again, the subsequent rinsing step caused no detachment of the formed S-layer lattices. As observed with SPR, a difference between the glycoprotein wtSgsE and the protein rSgsE was clearly detectable in both, the increase in areal mass density per time and the mass finally attached on the sensor surface. The increase in mass per time for rSgsE was faster and after approximately five minutes almost the entire mass of finally 789.4 ng cm^−2^ was attached to the sensor surface (Table 2). In contrast, the increase in mass per time was slightly slower for wtSgsE and there was an exponential approximation to the final mass of 893.9 ng cm^−2^ (Table 2), which was completed after a total time of approximately 25 min (Fig. 3B).

**Fig. 4:**
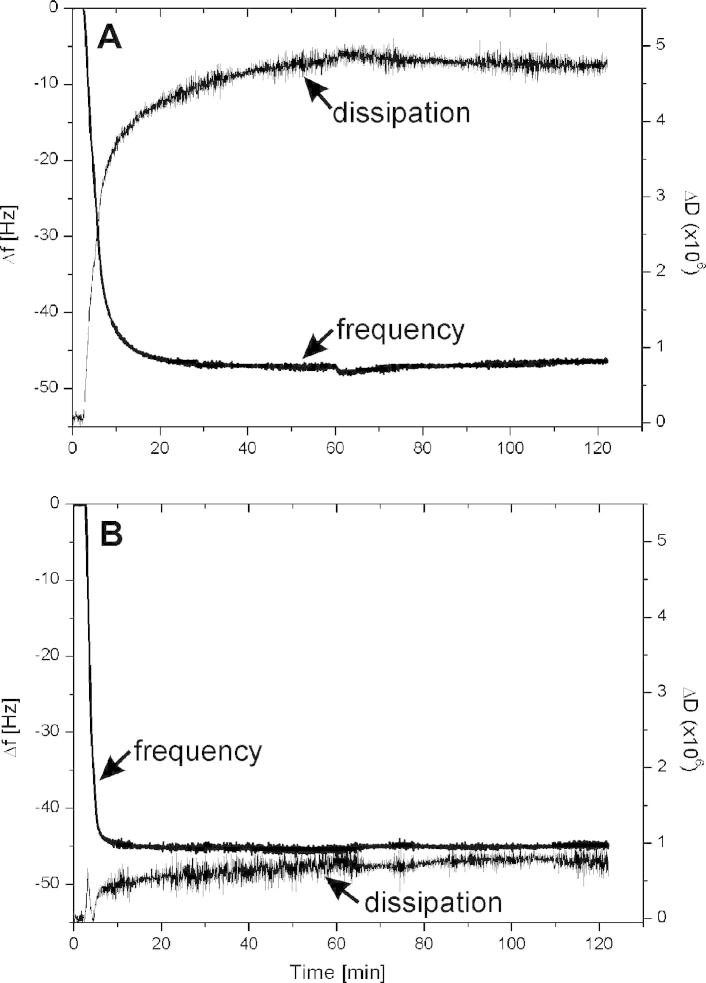
Recrystallization process of two different S-layer (glyco)proteins from *Geobacillus stearothermophilus* NRS 2004/3a (protein concentration of 50 μg mL^−1^) on gold-covered sensor surfaces as followed by quartz crystal microbalance with dissipation monitoring (QCM-D). The shift in frequency (left axis) and dissipation (right axis) as a function of time at the 5th overtone is shown for (A) the S-layer glycoprotein wtSgsE and (B) for the S-layer protein rSgsE.

In addition, QCM-D is also sensitive to changes in the viscoelasticity of the film adhering to the sensor crystal. These factors must be considered for the conversion of frequency shifts to mass change [42]. Besides the increase in mass (Fig. 3B), also the change in dissipation has been determined for wtSgsE and rSgsE at a concentration of 50 μg mL^−1^ (Fig. 4). Interestingly, for the glycoprotein wtSgsE the shift in dissipation was large compared to rSgsE and an exponential approximation to the final value of 4.5 × 10^−6^ was determined (Table 1; Fig. 4A). In contrast, the shift in dissipation was much smaller for the S-layer protein rSgsE as a final value of 0.79 × 10^−6^ was observed (Table 1; Fig. 4B).

Laterally homogeneous films may either induce a small shift in dissipation (rSgsE) or a large one (wtSgsE). A common rule of thumb is that, if Δ*D_n_*/(−Δ*f_n_*/*n*) ≪ 4 × 10^−7^ Hz^−1^ (for a 5 MHz crystal), then the film can be approximated as rigid, and the Sauerbrey equation [43] can be used to extract the areal mass density of the film [41,42]. Following this approximation, the Sauerbrey relation can be used for the evaluation of the rSgsE layer (Δ*D_n_*/(−Δ*f_n_*/*n*) ≈ 0.2 × 10^−7^ Hz^−1^) but also for the wtSgsE lattice (Δ*D_n_*/(−Δ*f_n_*/*n*) ≈ 0.9 × 10^−7^ Hz^−1^). Moreover, the data obtained for the wtSgsE layer with the Voigt-model (often used for the evaluation of highly viscoelastic thin films) and those from the Sauerbrey relation resulted within the standard deviation in the same values. Hence, throughout the whole study the Sauerbrey relation was used to calculate the areal mass density from the measured shift in frequency (Figs. 3B and 4).

Three likely candidates for the shifts in *D*, Δ*D*, are dissipation (i) at the protein–substrate interface, (ii) at the protein–liquid interface, including effects of a change of surface roughness, and (iii) within the protein layer (including effects of trapped liquid) [42]. Generally, in the case of laterally homogeneous films, the dissipation occurs inside the film and is related to the viscoelastic properties of the material [44]. In contrast, in the case of laterally heterogeneous films composed of, *e.g.*, discrete particles, most of the dissipation occurs at the liquid-particle boundary and is related to the properties of the particle-surface contact region [41]. However, as both S-layer lattices are homogeneous layer (see Fig. 2) and in both cases the protein portion of the S-layer is directly in contact with the gold surface without any intermediate linkers, no contribution to the dissipation from the S-layer-gold electrode region can be expected. It is much more conceivable that the high dissipation value for wtSgsE reflects an elevated viscoelasticity caused by the elongated glycan portion.

**Fig. 5:**
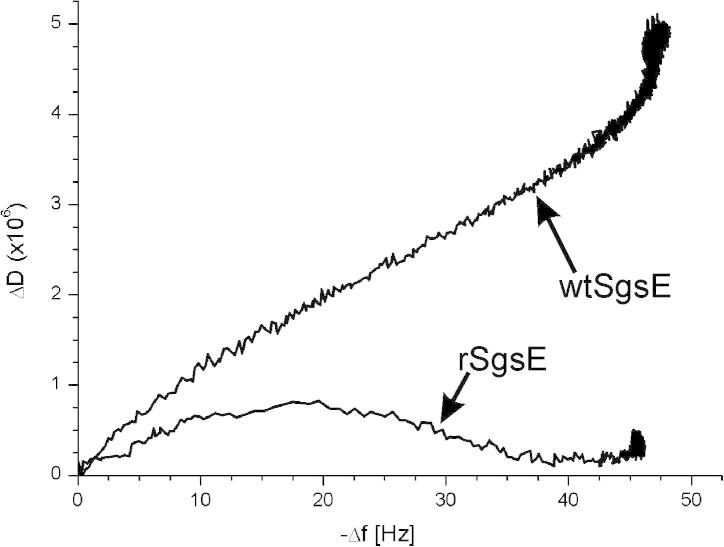
Frequency – dissipation curves upon the recrystallization process of the S-layer glycoprotein wtSgsE from *Geobacillus stearothermophilus* NRS 2004/3a and its truncated analog, the S-layer protein rSgsE at a protein concentration of 50 μg mL^−1^.

**Fig. 6:**
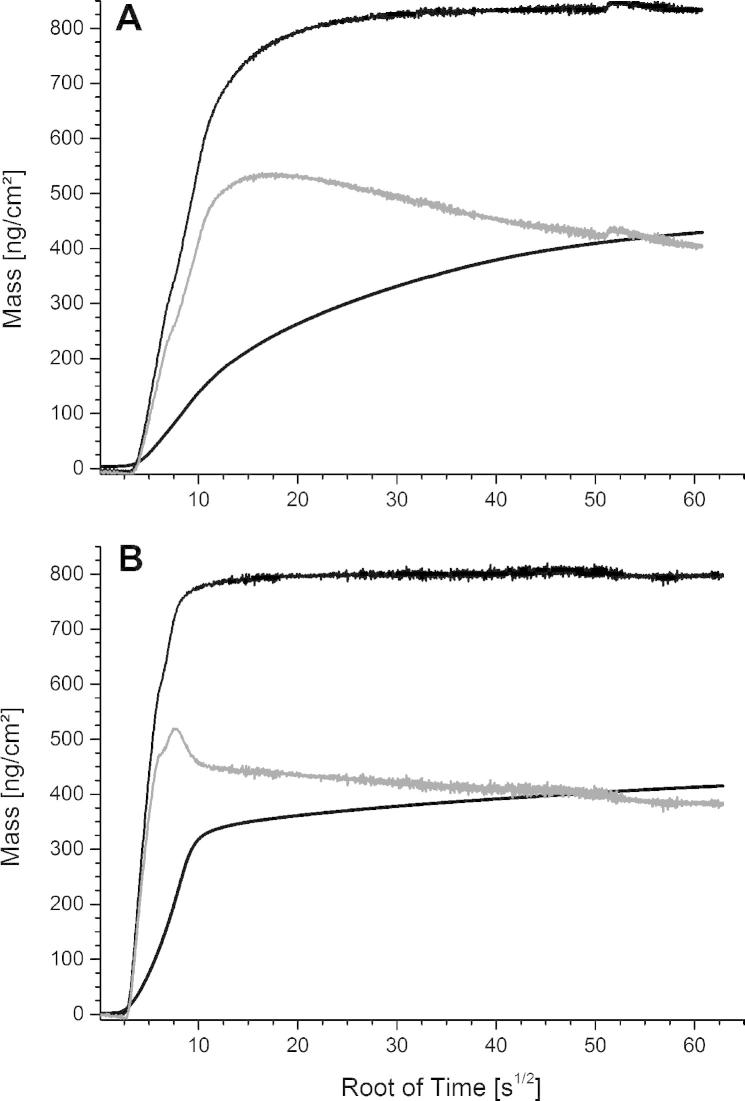
The progress of areal mass density of the biomolecule as determined by SPR (lower black trace), the biomolecule with dynamically coupled and bound water as determined by QCM-D (upper black trace) and the thus calculated difference representing the areal mass density of the dynamically coupled and bound water (intermediate gray trace) for (A) the S-layer glycoprotein wtSgsE from *Geobacillus stearothermophilus* NRS 2004/3a and (B) its truncated analog, the S-layer protein rSgsE.

The frequency–dissipation curves (Fig. 5) qualitatively illustrate the link between the stages of recrystallization and the mechanical properties of the layer. QCM-D monitoring provides additional information on how the viscoelastic structure of the S-layer protein changed per unit mass attachment. For wtSgsE, an almost linear increase was observed up to a dissipation value of approximately 4.0 × 10^−6^, subsequently the dissipation increased to 4.5 × 10^−6^, although the increase in mass was rather low (see Figs. 4A and 5). Hence, the increase in areal mass density was accompanied by a linear increase in softness. A possible explanation is that, once the system had almost reached maximal coverage, large glycoprotein domains transformed into the final, low-energy, stable state [6,7], which was accompanied by an increase in dissipation before completion. In contrast, rSgsE showed a different behavior as there was an increase in dissipation to a maximum of approximately 0.8 × 10^−6^ up to a shift in frequency of −20 Hz and subsequently the dissipation dropped down with increasing mass (decreasing frequency; Fig. 4B) to a constant value from a shift in frequency of approximately −40 Hz (Fig. 5). From this behavior one can conclude that in the first stage of the adsorption and self-assembly process the rSgsE layer showed a moderate rigidity, most probably because water is between the initially formed crystalline domains [6,7], and subsequently, the S-layer lattice became even more rigid with increasing mass due to the formation of a solid-like, coherent proteinaceous layer. This assumption is further supported by analyzing the areal mass density of (dynamically) coupled and bound water with respect to the elapsed time (see Fig. 6, next Section).

Up to now, most QCM-D studies have been performed with the S-layer protein SbpA from *Lysinibacillus sphaericus* CCM 2177. The S-layer lattice is composed of identical non-glycosylated protein subunits (*M*_w_ of 127 kDa) forming a square (p4) lattice symmetry with a spacing of 13.1 nm between morphological units. The monomolecular lattice shows a thickness of approximately 9 nm [45]. If SbpA was recrystallized on the gold surface of a QCM-D sensor crystal, the frequency decreased to 84 ± 5 Hz, a value almost twice than that observed for wtSgsE and rSgsE and the dissipation raised up to a value of 2.2 ± 0.4 × 10^−6^ [46]. The non-glycosylated S-layer protein SbsB of *G. stearothermophilus* pV72/p2 is with respect to the lattice symmetry and constants (oblique, *a* = 9.4 nm, *b* = 7.4 nm, *γ* = 80°), molecular weight (approximately 100 kDa), and most probably also the thickness of the proteinaceous S-layer lattice of approximately 4.5 nm similar to SgsE [47]. When SbsB was recrystallized on the gold surface of a QCM-D sensor crystal, the frequency decreased by approximately 38 ± 2 Hz, a value close to that of rSgsE and the dissipation raised up to a maximal value of 1.1 ± 0.1 × 10^−6^ (*n* = 6). Hence, compared to the non-glycosylated S-layer proteins SbpA and SbsB but also to rSgsE, the glycoprotein wtSgsE showed a remarkable high dissipation value. Thus, this result strongly supports the previously made assumption that the high dissipation value for the recrystallized wtSgsE reflects an elevated viscoelasticity of the glycan portion because all non-glycosylated S-layer proteins including rSgsE revealed a rigid layer displaying a low dissipation value particularly if set into relation to the shift in frequency.

### 3.5 Comparison of the data measured by SPR and QCM-D

Due to the fact that with SPR as an optical method the surface-attached mass of the biomolecules (in this case protein or protein and attached carbohydrate moieties for rSgsE and wtSgsE, respectively) is measured and QCM-D as acoustical method detects not only the attached biomolecules but also the bound and coupled mass of water, the determined values for the areal mass density are generally larger measured by QCM-D compared to SPR. Indeed, for wtSgsE the determined areal mass density of the entirely covered sensor area was 439.2 ng cm^−2^ and 893.9 ng cm^−2^ and for rSgsE 402.5 ng cm^−2^ and 789.4 ng cm^−2^ measured by SPR and QCM-D, respectively (Table 2). The difference between the QCM-D mass and the SPR mass can be attributed to the mass related to the bound and coupled mass of water. The latter was 454.7 ng cm^−2^ and 386.9 ng cm^−2^ for wtSgsE and rSgsE, respectively after the recrystallization process has been finished. The ratio of mass of water divided by mass of biomolecules (protein with or without glycans) is calculated for both to be approximately one.

Generally speaking, the same tendency for the increase in areal mass density per time and the mass finally attached on the sensor surface was observed for wtSgsE and rSgsE with both surface sensitive methods. The biomolecular surface was controlled at final mass areal density by AFM and it could be confirmed that a coherent S-layer lattice comprising of wtSgsE and rSgsE, respectively has been recrystallized on the sensor surfaces. Because of the higher molecular mass of wtSgsE compared to rSgsE, the higher measured final mass deposited per unit area is conceivable. The much slower (SPR) and significantly slower (QCM-D) increase in mass per unit area and exponential approximation to the final areal mass density for wtSgsE (Fig. 3A) was not anticipated. For instance, for two truncated forms of the non-glycosylated S-layer protein SbpA from *L. sphaericus* CCM 2177, the SbpA_31–318_ (*M*_r_ ≈ 31 kDa) and the SbpA_31–1068_ (*M*_r_ ≈ 110 kDa), almost the same increase in mass deposited per unit area in time was determined by QCM-D [48]. Hence, one may speculate that not the difference in molecular mass between wtSgsE and rSgsE is mainly responsible for the slower increase in mass per unit area and exponential approximation to the final areal mass density for wtSgsE. In fact, there might be a contribution of the glycan residues on the C-terminus of wtSgsE and the outcome of this is a different biomass to water ratio. Interestingly, in approximately the first third of the increase in mass per unit area there is almost no difference between wtSgsE and rSgsE measured with both, SPR (Fig. 3A) and QCM-D (Fig. 3B). Thus, there is no difference in the initial surface attachment (adsorption) for both S-layer (glyco)proteins but later on, the glycan residues might decelerate the subsequent self-assembly and recrystallization process of wtSgsE.

Valuable information is given by plotting the areal mass density per root of time obtained by SPR and QCM-D to determine the areal mass density in time response of the dynamically coupled and bound water (Fig. 6). For both S-layer proteins it can be seen, that the mass area density of the water increased to a maximum and subsequently declined to a final, stable equilibrium value. Interestingly, for rSgsE a sharper peak with a higher maximal mass area density was observed compared to the curve progression of wtSgsE. This finding can be explained besides the water surrounding the protein and glycoprotein, respectively, by trapped water between the initially formed crystalline S-layer domains. But in the course of the recrystallization of rSgsE the crystalline domains meet each other to form a coherent lattice on the sensor surface and hence, the trapped water is squeezed out. This pronounced squeezing-out process cannot be observed at wtSgsE, thus pointing to differences caused by the formation of nucleation points. In addition, the glycan residues might decelerate the squeezing out effect of the bound water into the bulk (Fig. 6). This finding is also supported by plotting the water to rSgsE and wtSgsE mass, respectively in dependence of the root of time (see Supplementary data, Fig. S2) [49].

Moreover, the present study demonstrates that, in line with previously reported findings [49], QCM-D monitoring without using a further surface sensitive method can hardly be used to follow the kinetics of the adsorption, self-assembly and recrystallization process of, *e.g.*, proteins by measuring the mass deposited per unit area. The reason is that the change in water content sensed by QCM-D with respect to the protein mass area density will not be taken into account which gives a biased kinetics of the process.

Interestingly, the difference in adsorbed material for wtSgsE and rSgsE when calculated from the QCM-D data is with 104.5 ng cm^−2^ almost 3 times the value compared to that retrieved from SPR measurements (36.7 ng cm^−2^) (Table 2). Again, the difference between the QCM-D mass and the SPR mass should give the mass related to the bound and coupled mass of water (which was calculated to be 67.8 ng cm^−2^). However, in the present case the ratio between the excess biomass (130 amino acids and glycan residues) and water was calculated to be approximately 1:2 indicating that almost twice the amount of water is bound and coupled to that part of the biomass where wtSgsE differs from rSgsE. A preferred region where water could in particular be bound and/or coupled might be the outermost glycan residues of the wtSgsE layer. In a recent study it has been reported that the glycan residues of the surface-adsorbed glycoproteins apparently impose a long-range ordering on the vicinal water [50]. These results indicate that the glycans in the cell membrane may play a decisive role in the ordering of water, which could influence the behavior of biomolecules that move into this vicinal water, via interactions with their hydration layers. On the other hand it has been shown that the amount of water sensed by QCM-D was found to increase with the nano-roughness of the coupled layer on the sensor [51], a parameter which could be different for wtSgsE and rSgsE, respectively (Fig. 1). The glycan chains are bound with one end on the protein and experience a certain movement and may be visualized as weed in the sea. From a statistical point of view, a folding back of the glycan chains toward the protein lattice is entropically improbable whereas the upright, protruding state is the most supposable position of the glycan moiety (P. Kosma, pers. commun.) as it is indicated in Fig. 1A. In this case, not only the QCM-D mass loading would be increased but also the dissipation [52]. Indeed, this is exactly what we have observed (see Table 1). Thus, although it could not be distinguished by AFM, the hydrated wtSgsE layer may possess a higher surface nano-roughness and thus, an altered fluidic mechanics in the nanoscale due to the water-exposed glycans.

### 3.6 Influence of the protein concentration on the QCM-D data

The values for the frequency and dissipation, taken after completion of adsorption/recrystallization and subsequent rinsing *i.e.*, when no further changes in frequency and dissipation are observed were plotted as a function of S-layer protein concentration (see Supplementary data, Fig. S3). For both S-layer (glyco)proteins the shift in frequency increases with concentration until a plateau is reached at a concentration of 50 μg mL^−1^. Except for the value of 10 μg mL^−1^ of wtSgsE, the shift in frequency was higher for wtSgsE compared to rSgsE (Fig. S3A). Concerning the shift in dissipation, again 50 μg mL^-1^ but only 20 μg mL^−1^ were necessary to achieve the maximal value for wtSgsE and rSgsE, respectively. The shift in dissipation was generally higher for wtSgsE compared to rSgsE.

The effect of the protein concentration on the adsorption behavior shows that the same amount, *i.e.*, 50 μg mL^−1^ of wtSgsE and rSgsE, respectively is necessary to reach a constant Δ*f* value on the gold-coated sensor surfaces, indicating that the S-layer protein affinity for the sensor surfaces is similar. The effect of the protein concentration on the viscoelastic property shows that a higher amount of 50 μg mL^−1^ is necessary to reach a constant Δ*D* value for wtSgsE but the Δ*D* value remains almost constant up to a concentration of 20 μg mL^−1^ for rSgsE (see Supplementary data, Fig. S3B). This finding indicates that a lower amount of rSgsE compared to wtSgsE forms a rigid layer on the sensor surface whose rigidity is not further influenced by increasing the concentration of rSgsE.

### 3.7 Determination of the specific density and thickness of the re-crystallized S-layer lattices

The specific density and thickness of the recrystallized rSgsE and wtSgsE layers have been calculated (see Supplementary data, Eq. (1)). The specific density of rSgsE is with 1.18 g cm^−3^ slightly lower than those calculated for wtSgsE having a value of 1.21 g cm^−3^ (Table 1). This result was expected as the specific density of, *e.g.*, polysaccharides or the α-1-acid glycoprotein (AGP) is with *ρ* = 1.65 g cm^−3^ and *ρ* = 1.42 g cm^−3^, respectively higher compared to the specific density usually taken for proteins (*ρ* = 1.35 g cm^−3^) [50]. In previous studies, the specific density for the truncated S-layer protein SbpA_31–1068_ and native SbpA has been calculated to be 1.12 g cm^−3^ and 1.14 g cm^−3^, respectively [48,53]. Hence, not only for all S-layer proteins but also for the wtSgsE a lower specific density as the experimental determined value of 1.35 g cm^−3^ for proteins (*e.g.*, the enzyme malate dehydrogenase) has been obtained [54]. This seems to be a special feature of the porous S-layer (glyco)proteins [1,2].

The “hydrated” thickness of the truncated S-layer protein rSgsE and the glycoprotein wtSgsE was calculated to be 6.8 nm and 7.6 nm, respectively (Table 1) (see Supplementary data, Eq. (2)). The data for wtSgsE are in good agreement with those of a previous study where the apparent thickness was found to be 7–8 nm as judged from contrast-related maximum–minimum curves in 3D data sets [40]. However, by this method the obtained three-dimensional structure is stretched in the *z*-direction and thus, the thickness is known to be estimated slightly too long [55]. Hence, the here calculated thickness and the previously reported one match pretty well with each other. The difference in thickness between wtSgsE and rSgsE is 0.8 nm, which is only a fractional amount of the calculated length of the glycan residues of 32 nm. Possible explanations might be that wtSgsE is to a relatively low content glycosylated (3.3%) and hence, as SPR and QCM-D average the glycan mass over the whole area, the calculated thickness is much too short. On the other hand, it cannot be excluded, that individual glycan antennae were not extending up, but rather were covering the peptide backbone and rendering the liquid-glycoprotein interface hydrophilic as it was observed for, *e.g.*, AGP [50].

## 4 Conclusions

We have investigated for the first time the recrystallization and surface properties of the wild-type S-layer glycoprotein wtSgsE from *G. stearothermophilus* NRS 2004/3a and its recombinantly produced non-glycosylated form rSgsE on gold sensor surfaces. Both S-layer (glyco)proteins revealed a similar, laterally homogeneous morphology with a higher hydrated thickness for wtSgsE compared to rSgsE (Table 1). As expected from the molecular weights and composition, a higher specific density (Table 1) and areal mass density (Table 2) were observed for wtSgsE and rSgsE, respectively. The final ratio of mass of water divided by mass of biomolecules (protein with or without glycans) is calculated for both to be approximately one. The remarkable high dissipation compared to all S-layer proteins studied so far indicated an elevated viscoelasticity for wtSgsE, which can most probably be attributed to the glycan portion. Moreover, almost twice the amount of water was bound and/or coupled to the outermost glycan residues of the wtSgsE layer (Table 2). Interestingly, it has been shown that the self-cleaning capability of biological surfaces relies not only on the wettability but rather on the structure of the water molecules near the substrate [56]. Hence, one may speculate that the infiltration of the nanoporous S-layer lattice with lubricating water might exhibit some kind of S-layer specific “nano-pitcher-plant effect” [57].

As a result from this study and previously published data one may presume on the common intrinsic features of S-layer lattices as biocompatible, antifouling and/or self-cleaning structure due to the combination of polyzwitterionic and polyhydrophilic characteristics, nanopatterned interface with the roughness in the nanometer scale, and a locked-in-place hydration layer facing the surface [27,28]. The latter can even be modulated by the presence of surface-exposed glycan residues. However, further studies are necessary to clarify this point as in contradiction to increased slippage a relocate-and-seek behavior enabling archaeal cells to seek for and remain in a favorable surrounding has been reported [33].

In summary, this general feature of S-layers could explain the widespread occurrence of these monomolecular arrays in the world of prokaryotic organisms and justify the energy cost for their synthesis. This general feature is not in contradiction with more specific functional properties observed, *e.g.* in pathogenic organisms or specific cell tissue interactions [24]. Since S-layer (glyco)protein subunits reveal the intrinsic property to recrystallize as a coherent layer on a great variety of surfaces and interfaces [1,3,6], they might exhibit a biomimetic application potential for specific surface modifications such as antifouling coatings, biocompatible surfaces, biosensors, diagnostics, drug targeting and delivery systems or microfluidics-guided applications.

## Disclosures

The authors declare no conflicts of interest.

## Figures and Tables

**Table 1:** Summary of the intrinsic properties of wtSgsE and rSgsE and their recrystallized monolayers entirely covering the gold-coated sensor surfaces (SgsE concentration: 50 μg mL^−1^ each; ^∗^*n* = 5).

	wtSgsE	rSgsE
Molecular weight [Da]	97,670	82,800
Protein content [%]	97.7	100
Glycan content [%]	3.3	0
Shift in frequency [Hz]	50.5 ± 4.1^∗^	44.6 ± 0.6^∗^
Shift in dissipation (×10^−6^)	4.50 ± 0.18^∗^	0.79 ± 0.03^∗^
Thickness [nm]	7.6	6.8
Specific density [g cm^−3^]	1.21	1.18

**Table 2:** Summary of the final mass area density (*n* = 5) determined by SPR and QCM-D for recrystallized wtSgsE and rSgsE monolayers (SgsE concentration: 50 μg mL^−1^ each) entirely covering the gold-coated sensor surfaces (see also Fig. 3).

A wtSgsE [ng cm^−2^]	B rSgsE [ng cm^−2^]	Method	Remark
439.2 ± 9.5	402.5 ± 12.4	SPR	*n* = 4
893.9 ± 72.6	789.4 ± 10.6	QCM-D	*n* = 5
454.7	386.9	QCM-D, SPR	QCM-D–SPR
36.7		SPR	A–B
104.5		QCM-D	A–B
67.8		(QCM-D: A–B)–(SPR: A–B)	

## Appendix A Figures with essential color discrimination

Certain figures in this article, particularly Figs. 1 and 2 are difficult to interpret in black and white. The full color images can be found in the on-line version, at http://dx.doi.org/10.1016/j.actbio.2015.03.020.

## Appendix B Supplementary data

Supplementary data This document contains supplementary information.
