# Supplementary material for: Relevance of glycosylation of S-layer proteins for cell surface properties
Source: Acta Biomater. 2015 Jun;19:149–57. doi: 10.1016/j.actbio.2015.03.020 (PMC4414373; doi:10.1016/j.actbio.2015.03.020)
Supplement: Supplementary data — This document contains supplementary information. [file mmc1.docx]

**Supplementary data**

Relevance of glycosylation of S-layer proteins for cell surface properties

Bernhard Schuster^1*^ & Uwe B. Sleytr^2*^

^1^ Institute for Synthetic Bioarchitectures; ^2^ Institute for Biophysics

Department of NanoBiotechnology, University of Natural Resources and Life Sciences, Vienna; Muthgasse 11, 1190 Vienna, Austria.

*Corresponding authors: Bernhard Schuster, E-mail: bernhard.schuster@boku.ac.at; Fax: +43 1 4789112; Phone: +43 1 47654 2213. Uwe Sleytr, E-mail: uwe.sleytr@boku.ac.at; Fax: +43 1 4789112; Phone: +43 1 47654 2201.

**Preparation of the wild-type S-layer glycoprotein samples**

Wild-type S-layer glycoprotein was isolated from cell wall preparations by extraction with 5 M guanidine hydrochloride (GHCl; Fluka, Buchs, Switzerland) according to a previously described procedure.[1] The lyophilized protein was further purified on a Sephacryl S-200 column (1.6 by 60 cm; GE Healthcare, Uppsala, Sweden) using 2 M GHCl in 50 mM Tris-HCl buffer, pH 7.5, as eluent. After extensive dialysis, first against 15 mM CaCl_2_ and then against distilled water, the protein was lyophilized and stored at -20°C.

Purification of the wild-type protein was performed and monitored by sodium dodecyl sulfate polyacrylamide gel electrophoresis (SDS-PAGE; see Fig. S1) as previously described.[2] SDS-PAGE and visualization of proteins bands with Coomassie Blue R-250 staining reagent was carried out as previously described.[3] Detection of the band intensities of the wild-type SgsE (wtSgsE) was done with the Li-Cor Odyssey infrared imaging system. The integrated intensity of detected bands was determined using the Li-Cor Odyssey Application software 3.0.21. Protein concentration was determined by the Bio-Rad protein assay (Bio-Rad, Vienna, Austria) using bovine serum albumin (BSA) as standard.

**Surface plasmon resonance (SPR) measurements**

The SPR signal is the result of the change of angle of incident light at a metallic interface depending on the refractive indices of adsorbed materials. The geometrical thickness (Δd) of the adsorbed material can be deduced from the angle shift (ΔΘ) if the refractive index (Δn) of each layer is known. Taken together, an angle shift of ΔΘ = 0.1° corresponds to a protein surface concentration of 1 ng/mm² or to a thickness of 10 Å.[4] The change of the SPR signal is given in resonance units (RU), a dimensionless quantity that is proportional to the change in refractive index, (Δn, at the interfacial region according to the BIAtechnology handbook (BIAcore AB), where 1000 RU are equivalent to 1 ng protein per mm². Thus, when calibrated for adsorption of proteins on flat surfaces,[5, 6] SPR allows real-time measurements of mass uptake (Δm_SPR_).

**Quartz crystal microbalance with dissipation monitoring (QCMD) measurements**

QCM-D measurements were carried out with a QE401 (electronic unit)/QFM401 (flow module) instrument from Q-sense AB (Gothenburg, Sweden). A brief technical description about this technique has been given by Rodahl et al., 1997.[7] Before the recrystallization of the recombinant or wildtype SgsE, the crystal was rinsed with Milli-Q water containing 5 mM calcium chloride (solution A) with a flow rate of 50 mL min^-1^ until a constant baseline was achieved. The solutions of SgsE proteins in solution A with the given concentrations were passed over the sensor surface with a continuous flow of 50 mL min^-1^ until no further change in the signal was observed.

Protein binding and recrystallization were analyzed by the linear Sauerbrey relation as the protein layer is thin (> 25 nm) and more or less rigid and thus, the decrease in frequency is proportional to the mass of the hydrated protein (Δm_QCM-D_).[8] The data (shift in frequency (Δf) and in dissipation (ΔD) at the overtones n = 3, 5, 7, 9, 11, 13) measured at the recrystallization of the wildtype SgsE on the gold surface have also been fitted to the Voigt-based viscoelastic model as the Sauerbrey relation may fail in thickness calculation for soft films.[9] This fitting routine is included in the Q-Tools software. However, it turned out, that there was no significant difference between the data calculated by the Sauerbrey relation and the Voigt-based viscoelastic model. Hence, in this case, the Sauerbrey equation can be used to estimate the adsorbed areal mass density and the wet thickness of the S-layer lattice can be roughly estimated from Δm_QCM-D_/ρ_SLP_ by assuming a homogeneous S-layer lattice with the specific density ρ_SLP_ of the S-layer protein given by Espinosa-Marzal et al., 2013:[10]

ρ_SLP_ = (Δm_QCM-D_ - Δm_SPR_) × ρ_solv_ / Δm_QCM-D_ + Δm_SPR_ × ρ_biomol_ / Δm_QCM-D_ (1)

where ρ_biomol_ is the density of the S-layer protein (~1.35 g cm^-3^) or glycoprotein (~1.42 g cm^-3^), respectively[10] and ρ_solv_ is the specific density of the Milli-Q water (0.997 g cm^-3^ at 25 °C).

Moreover, the thickness of the recrystallized S-layer lattice can be readily estimated by the following equation (2).[11]

h_SLP_ = 1/ρ_solv_ × (Δm_QCM-D_ - Δm_SPR_ × (1- ρ_solv_ /ρ_biomol_ )) (2)

**Estimation of the composition of the glycoprotein wtSgsE from *Geobacillus stearothermophilus* NRS 2004/3a**

From the SDS-PAGE (Fig. S1), the integrated intensities of the bands corresponding to non-glycosylated wtSgsE and the glycoprotein species where one, two or three glycosylation sites are occupied by glycan chains were estimated. This analysis resulted in a composition of the wtSgsE of 66.2 ± 5.1% non-glycosylated, 24.2 ± 3.6% carrying one glycan chain, 7.7 ± 1.9% carrying two glycan chains, and 1.9 ± 1.3% carrying three glycan chains. According to a previous study, the respective molecular weights, M_r_, are M_r_ = 94,460 Da, M_r_ = 101,660 Da, M_r_ = 108,680 Da, and M_r_ = 115,730 Da, respectively.[1, 2] In comparison to SDS-PAGE analysis, where the migration behaviour of glycoproteins on the gel is frequently influenced by the attached glycan chains, in the study of Steiner et al.[2] MS analysis was used as this is a more reliable alternative for accurate mass determination of glycoproteins.[12, 13]

| 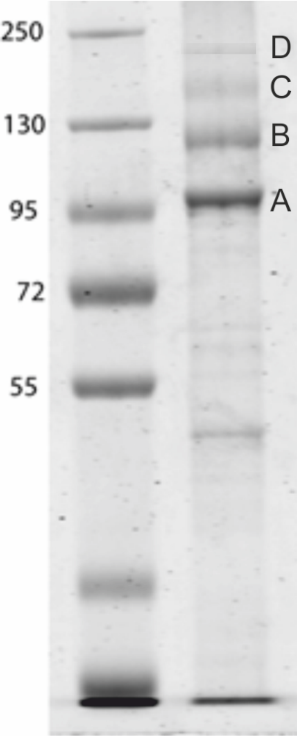 | Fig. S1: SDS-PAGE (sodium dodecyl sulfate-polyacrylamide gel electrophoresis) analysis (8% gel) after Coomassie Blue R-250 staining of the S-layer glycoprotein wtSgsE of *G. stearothermophilus* NRS 2004/3a. Lane 1: Bench-mark ladder (Invitrogen); lane 2: purified wtSgsE glycoprotein showing four bands corresponding to non-glycosylated wtSgsE (A) and the glycoprotein species where one (B), two (C) or three (D) glycosylation sites are occupied by glycan chains. |
| --- | --- |

Moreover, the average mass of the homopolymeric l-rhamnan found in the S-layer glycan of *G. stearothermophilus* NRS 2004/3a was reported to be 7,090 Da.[2, 14] Thus, the composition of the wtSgsE can be estimated on the basis of the composition determined above. This calculation resulted in a mass for the glycan portion of 3,210 Da, which corresponds to approximately 3.3% of the mass of wtSgsE and 96.7% S-layer protein with a mass of 94,460 Da.[2] From these data, the mean molecular weight of the used wtSgsE can be calculated to 97,670 Da. The molecular weight of the non-glycosylated, truncated rSgsE was calculated to 82,800 Da.[15] The degree of glycosylation of the S-layer protein wtSgsE with 3.3% is in good accordance with previously published data where it has been reported that the degree of glycosylation of bacterial S-layer proteins, that is, the covalent *O*-glycosidic linkage of glycan moieties to select serine, threonine, and tyrosine residues, varies generally between 2% and 10% (w/w).[16, 17]

**Estimation of the glycan residue length of the glycoprotein wtSgsE**

The molecular length of the branched, fully extended homopolymeric glycan chain composed of 15 tri-rhamnose repeating units was estimated to approximately 32 nm by assuming 0.49 nm for the maximal radius of the l-rhamnose residue.[18] Hence, the length of the extended glycan chain is approximately four times higher than the thickness of the wtSgsE monolayer. This value is also in good accordance with ferritin labelling of the glycan chains of the S-layer glycoprotein from *Clostridium thermohydrosulfuricum* L111-69.[19, 20]

**Water to mass ratio of rSgsE and wtSgsE in dependence of time**

Whereas in the initial phase of rSgsE adsorption/recrystallization more mass of water is associated with mass of rSgsE (a ratio of approximately 5), the mass of water decreases rather rapidly to a final ratio of approximately 1 (Fig. S2).


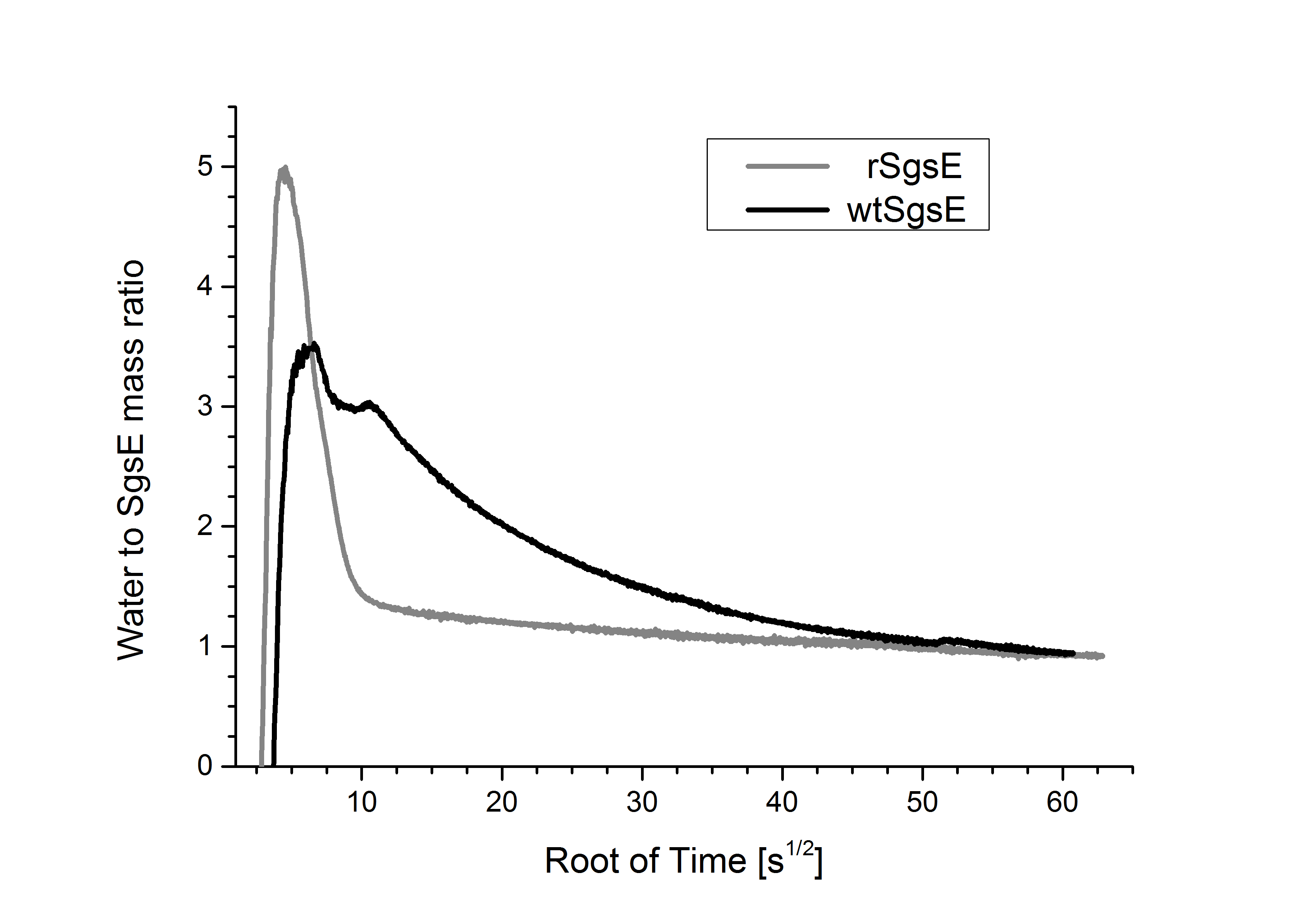


Fig. S2: Water to rSgsE and wtSgsE mass ratio in dependence of time (root of seconds). For more details concerning the calculation please see Reimhult et al., 2004.[21]

This finding might be explained by having first trapped water between the initially formed crystalline S-layer domains. But in the course of the recrystallization of rSgsE the crystalline domains meet each other to form a coherent lattice on the sensor surface and hence, the trapped water is squeezed out. In contrast, at wtSgsE the water to wtSgsE mass ratio showed two peaks. The first might point to differences in the formation of nucleation points between wtSgsE and rSgsE and hence the mass of water trapped between the crystalline domains meet. The second peak might appear because the glycan residues on wtSgsE might decelerate the squeezing out effect of the bound water into the bulk. This effect is also continued as the water to wtSgsE decreased slower comparted to rSgsE. However, finally the water to wtSgsE mass ratio decreased also to approximately 1, the same ratio as observed with rSgsE (Fig. S2).

**Influence of the protein concentration on the QCM-D data ~~and adsorption rate~~**

Figure~~s~~ S3 ~~and S4~~ shows the shift in frequency (A) and dissipation (B) in dependence of the biomass concentration. ~~graphically version of the data summarized in Table 2:~~


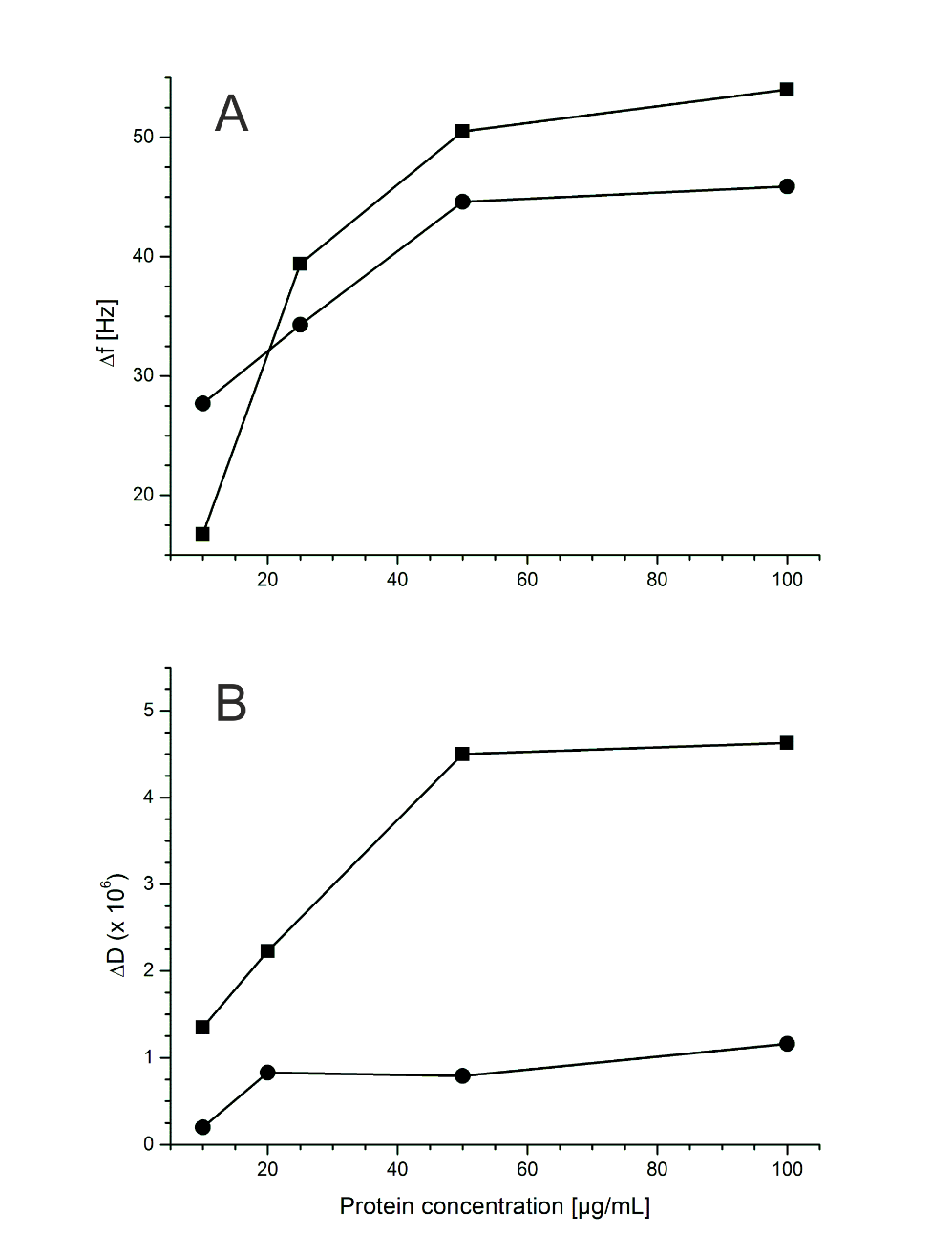


Fig. S3: Overall shift in (A) frequency and (B) dissipation at different concentrations of S-layer glycoprotein wtSgsE from *G. stearothermophilus* NRS 2004/3a (square symbol) and its truncated analogue, the S-layer protein rSgsE (circular symbol). In (A), ΔF increases for both types of SgsE with concentration until a plateau is reached at a concentration of 50 µg mL^‑1^. In (B), ΔD increases for both types of SgsE with concentration until a plateau is reached at a concentration of 50 µg mL^‑1^ and 20 µg mL^‑1^ for wtSgsE and rSgsE, respectively.


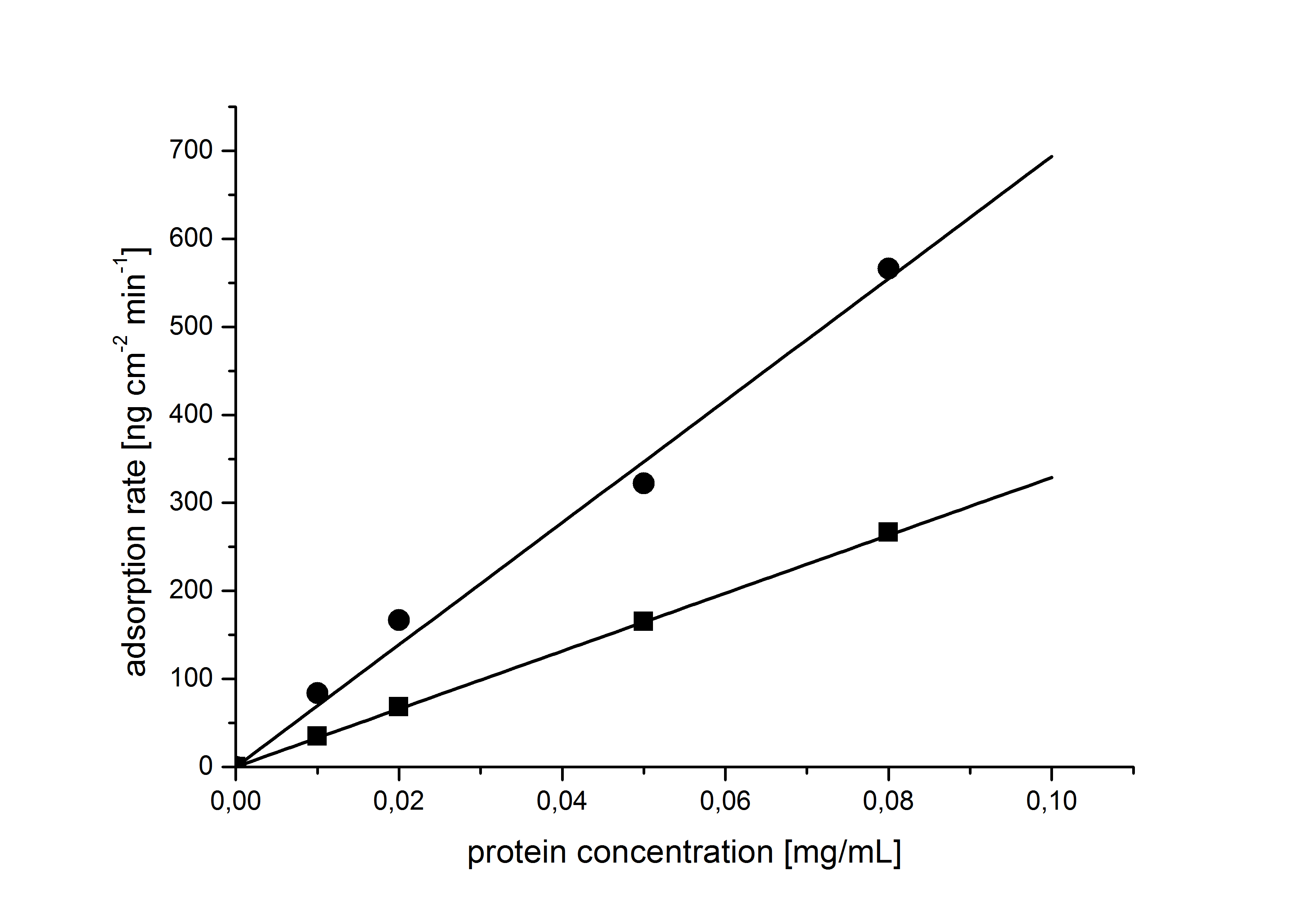


Figure S4 should be deleted

~~Fig. S4: Initial adsorption rate calculated from the derivative of frequency–time curves and at different concentrations of wtSgsE from~~ *~~G. stearothermophilus~~* ~~NRS 2004/3a (square symbol) and its truncated analogue, the S-layer protein rSgsE (circular symbol), respectively. A linear relationship is found, which suggests a diffusion-controlled process. Comparing the two slopes, it is observed that the adsorption rate is faster for rSgsE than for wtSgsE.~~

References

[1] Messner P, Pum D, Sleytr UB. Characterization of the ultrastructure and the self-assembly of the surface layer of *Bacillus stearothermophilus* strain NRS 2004/3a. J Ultrastruct Mol Struct Res 1986;97:73-88.

[2] Steiner K, Pohlentz G, Dreisewerd K, Berkenkamp S, Messner P, Peter-Katalinić J, et al. New insights into the glycosylation of the surface layer protein SgsE from *Geobacillus stearothermophilus* NRS 2004/3a. J Bacteriol 2006;188:7914-21.

[3] Altman E, Schaffer C, Brisson JR, Messner P. Characterization of the glycan structure of a major glycopeptide from the surface layer glycoprotein of *Clostridium thermosaccharolyticum* E207-71. Eur J Biochem 1995;229:308-15.

[4] Knoll W. Interfaces and thin films as seen by bound electromagnetic waves. Annual Review of Physical Chemistry1998. p. 569-638.

[5] Jonsson U, Fagerstam L, Ivarsson B, Johnsson B, Karlsson R, Lundh K, et al. Real-time biospecific interaction analysis using surface plasmon resonance and a sensor chip technology. Biotechniques 1991;11:620-2+4-7.

[6] Stenberg E, Persson B, Roos H, Urbaniczky C. Quantitative determination of surface concentration of protein with surface plasmon resonance using radiolabeled proteins. J Colloid Interface Sci 1991;143:513-26.

[7] Rodahl M, Höök F, Fredriksson C, Keller CA, Krozer A, Brzezinski P, et al. Simultaneous frequency and dissipation factor QCM measurements of biomolecular adsorption and cell adhesion. Faraday Discuss 1997;107:229-46.

[8] Sauerbrey G. Verwendung von Schwingquarzen zur Wägung dünner Schichten und zur Mikrowägung. Z Phys 1959;155:206-22.

[9] Voinova MV, Rodahl M, Jonson M, Kasemo B. Viscoelastic acoustic response of layered polymer films at fluid-solid interfaces: Continuum mechanics approach. Phys Scr 1999;59:391-6.

[10] Espinosa-Marzal RM, Fontani G, Reusch FB, Roba M, Spencer ND, Crockett R. Sugars communicate through water: Oriented glycans induce water structuring. Biophys J 2013;104:2686-94.

[11] Reviakine I, Johannsmann D, Richter RP. Hearing what you cannot see and visualizing what you hear: Interpreting quartz crystal microbalance data from solvated interfaces. Anal Chem 2011;83:8838-48.

[12] Harvey DJ. Proteomic analysis of glycosylation: Structural determination of N- and O-linked glycans by mass spectrometry. Expert Rev Proteomics 2005;2:87-101.

[13] Zaia J. Mass spectrometry of oligosaccharides. Mass Spectrom Rev 2004;23:161-227.

[14] Steiner K, Novotny R, Werz DB, Zarschler K, Seeberger PH, Hofinger A, et al. Molecular basis of S-layer glycoprotein glycan biosynthesis in *Geobacillus stearothermophilus*. J Biol Chem 2008;283:21120-33.

[15] Kainz B, Steiner K, Sleytr UB, Pum D, Toca-Herrera JL. Fluorescence energy transfer in the bi-fluorescent S-layer tandem fusion protein ECFP-SgsE-YFP. J Struct Biol 2010;172:276-83.

[16] Messner P, Egelseer EM, Sleytr UB, Schäffer C. Bacterial surface layer glycoproteins and “non-classical” secondary cell wall polymers. In: Moran A, Holst O, Brennan PJ, von Itzstein M, editors. Microbial Glycobiology: Structures, Relevance and Applications. San Diego: Elsevier; 2009. p. 109-28.

[17] Messner P, Schäffer C, Kosma P. Bacterial cell-envelope glycoconjugates. Advances in Carbohydrate Chemistry and Biochemistry. 2013. p. 209-72.

[18] Jenkins RT, Bell RA. Molecular radii of probes used in studies of intestinal permeability. Gut 1987;28:110-1.

[19] Messner P, Sleytr UB. Bacterial surface layer glycoproteins. Glycobiology 1991;1:545-51.

[20] Sára M, Küpcü S, Sleytr UB. Localization of the carbohydrate residue of the S-layer glycoprotein from *Clostridium thermohydrosulfuricum* L111-69. Arch Microbiol 1989;151:416-20.

[21] Reimhult E, Larsson C, Kasemo B, Höök F. Simultaneous surface plasmon resonance and quartz crystal microbalance with dissipation monitoring measurements of biomolecular adsorption events involving structural transformations and variations in coupled water. Anal Chem 2004;76:7211-20.
